# Recent Strategies to Combat Infections from Biofilm-Forming Bacteria on Orthopaedic Implants

**DOI:** 10.3390/ijms221910243

**Published:** 2021-09-23

**Authors:** Emérito Carlos Rodríguez-Merchán, Donald J. Davidson, Alexander D. Liddle

**Affiliations:** 1 Department of Orthopaedic Surgery, La Paz University Hospital, 28046 Madrid, Spain; 2 Osteoarticular Surgery Research, Hospital La Paz Institute for Health Research—IdiPAZ (La Paz University Hospital—Autonomous University of Madrid), 28046 Madrid, Spain; 3 Eastman Dental Institute, University College London, London WC1E 6BT, UK; donald.davidson.17@ucl.ac.uk (D.J.D.); a.liddle@imperial.ac.uk (A.D.L.); 4 MSk Lab, Imperial College London, London SW7 2AZ, UK

**Keywords:** biofilm, bacteria, biofilm-forming bacteria, orthopaedic implants, infection

## Abstract

Biofilm-related implant infections (BRII) are a disastrous complication of both elective and trauma orthopaedic surgery and occur when an implant becomes colonised by bacteria. The definitive treatment to eradicate the infections once a biofilm has established is surgical excision of the implant and thorough local debridement, but this carries a significant socioeconomic cost, the outcomes for the patient are often poor, and there is a significant risk of recurrence. Due to the large volumes of surgical procedures performed annually involving medical device implantation, both in orthopaedic surgery and healthcare in general, and with the incidence of implant-related infection being as high as 5%, interventions to prevent and treat BRII are a major focus of research. As such, innovation is progressing at a very fast pace; the aim of this study is to review the latest interventions for the prevention and treatment of BRII, with a particular focus on implant-related approaches.

## 1. Introduction

Orthopaedic implants are medical devices, used in both elective and emergency trauma surgery, which perform numerous functions including fracture stabilisation, deformity correction, joint reconstruction, and act as anchor points for soft tissues reconstruction [1]. Early orthopaedic implants were engineered to purely transmit and resist mechanical forces necessary to perform their function whilst remaining biologically inert; however, modern implants also have the ability, through the local delivery of molecules and surface coatings, to enhance bone healing and osseointegration whilst reducing the foreign body reaction and the risk of infection [1].

Implant-related infection occurs following approximately 5% of all elective and emergency orthopaedic procedures and is a serious surgical complication. Treatment often results in revision surgery, which has high levels of associated morbidity and financial costs; some cases result in amputation and death [2,3,4,5,6]. Once an infection colonises the implant, a sessile bacterial community is established called a biofilm; biofilm-related implant infections (BRII) are a considerable healthcare burden and account for approximately 65% of all healthcare bacterial infections [7].

Orthopaedic implant-related infection cannot be simply prevented by implant design and implant surface characteristics; instead, it must be considered in the wider context of prevention strategies including patient, surgical, and healthcare factors (Table 1); however, the implant surface has the potential to be the final line of defence against microbial attack [1].

**Figure 1:**
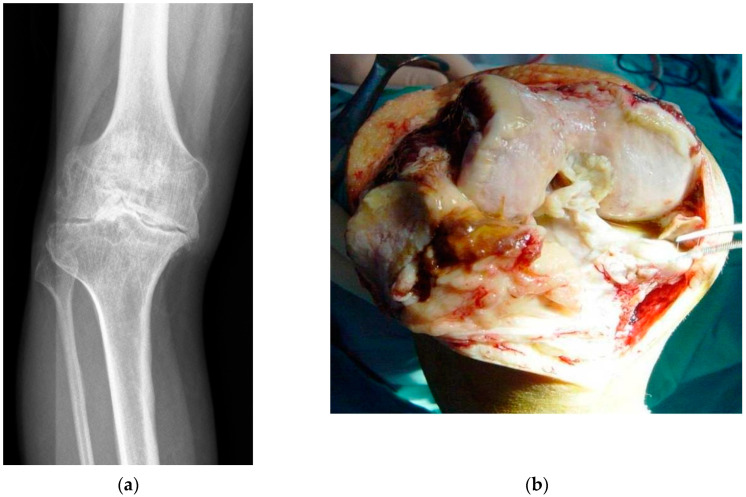

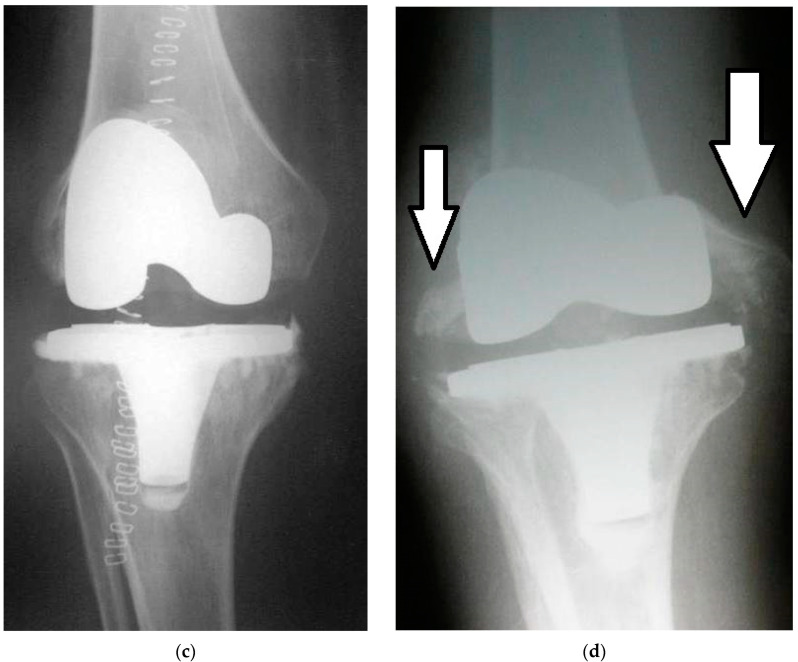
(**a**–**d**). *Staphyloccocus aureus* infection of a primary total knee arthroplasty (TKA): (**a**) Preoperative radiograph showing severe knee osteoarthritis; (**b**) Intraoperative image showing severe joint degeneration caused by osteoarthritis; (**c**) Immediate postoperative radiograph showing the implanted primary TKA (posterior stabilised cemented); (**d**) Radiographic image 5 months postoperatively showing severe osteolysis of the femoral condyles (arrows) due to periprosthetic joint infection (PJI). This patient required a two-stage revision arthroplasty in order to cure the infection.

**Figure 2:**
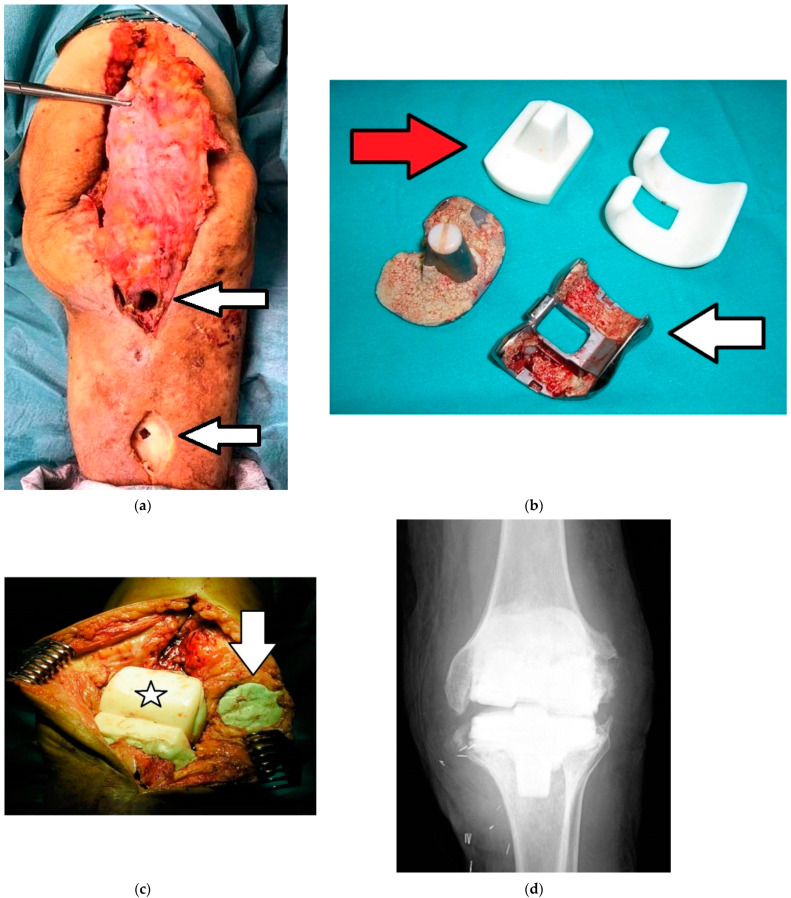
(**a**–**d**). Images of the same patient in Figure 1 relative to the first stage of the two-stage revision arthroplasty: (**a**) Image of the surgical field highlighting two fistulas caused by infection (arrows); (**b**) Femoral and tibial prosthetic components removed (white arrow) and antibiotic-loaded articulated spacer (red arrow) to implant; (**c**) Intraoperative image showing the articulated spacer already implanted (asterisk) and bone cement with gentamicin (green color) placed in the patellar component (arrow); (**d**) Postoperative radiograph showing the articulated spacer implanted.

**Figure 3:**
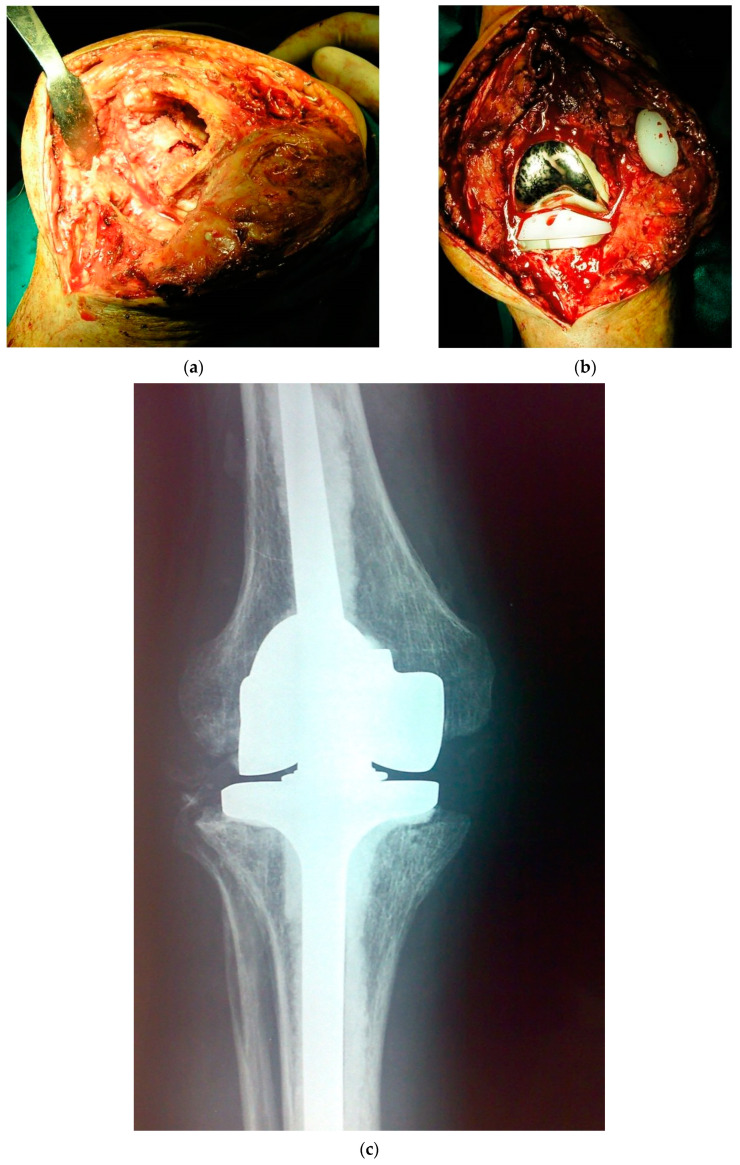
(**a**–**c**). Images of the same patient of Figure 1 and Figure 2 relative to the second stage of the two-stage revision arthroplasty: (**a**) Intraoperative image after removal of the articulated spacer; (**b**) Intraoperative image with the new prosthesis implanted; (**c**) Postoperative radiograph of the implanted prosthesis (rotating hinge total knee arthroplasty).

Direct inoculation of the surgical site can occur intraoperatively when bacteria enter from the local operating room environment (e.g., from the air or surfaces, surgical equipment, medical staff clothing) or the wound can be directly inoculated from bacteria found on the patient’s skin and body [1]. After surgical site contamination, the bacteria still need to adhere to the implant surface in order to establish a BRII; the “race to the surface” hypothesis proposed by Gristina, although simplistic, does allow the conceptualisation of the competition between the host’s cells and invading bacteria to adhere, replicate, and colonise the foreign surface of an implantable medical device [8,9] (Figure 1, Figure 2 and Figure 3).

BRII is clearly an important challenge in orthopaedic surgery with our understanding continually advancing and novel treatment strategies being developed. The purpose of this article is to review the most recent advances in strategies to combat infections due to biofilm-forming bacteria on orthopaedic implants. Following discussion of the biofilm and the bacterial species associated with it, we will discuss novel implant surface technology including passive and active surface modification; the use of local carriers and coatings, and biofilm eradication strategies.

For the purposes of this review, a bibliographic search was carried out on PubMed using the search string: [biofilm orthopedic implants], resulting in 817 articles, of which 142 papers were finally analysed because they were directly related to novel strategies to prevent and treat biofilm-forming bacteria on orthopaedic implants.

## 2. Bacterial Biofilm in Implant-Related Infections

**Figure 4:**
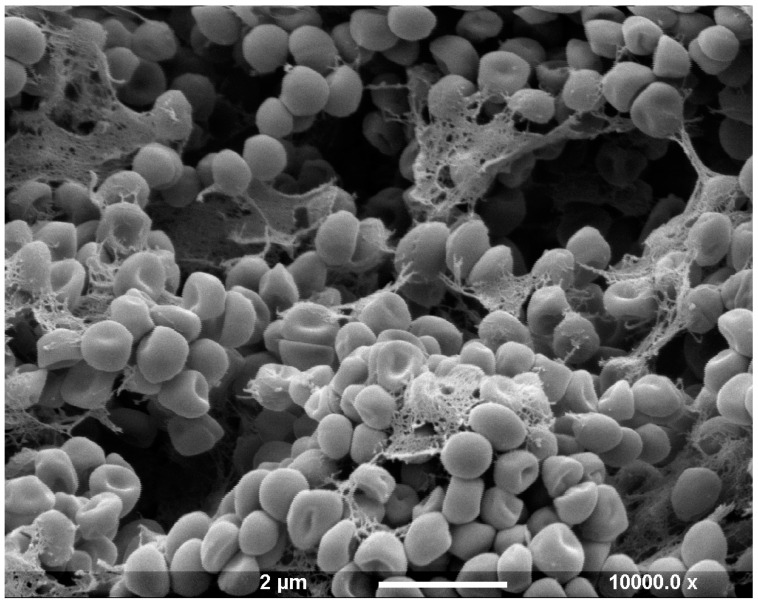
Scanning electron micrograph of a six-day-old Staphylococcus epidermidis biofilm demonstrating bacteria within an extracellular polymeric substance. Scale bar: 2 µm.

A bacterial biofilm is a colony of sessile bacteria irreversibly anchored to the implant surface and contained within a self-produced matrix known as extracellular polymeric substance (EPS), containing mainly polysaccharides, lipids, proteins, and extracellular DNA [10,11] (Figure 4).

In nature, 99% of bacteria exist inside biofilms, and the constituent biofilm bacteria exhibit an altered phenotype, growth rate, and gene transcription compared to planktonic, free-floating bacteria in suspension [10,11,12]. Bacterial biofilms display emergent properties: that is, properties that are not predictable from study of the planktonic cells from which they emerge [12]. These properties give the biofilms a significant survival advantage, making them extremely resilient to host immune or conventional anti-microbial therapies; they have been shown to be up to 1000× more resistant to antibiotic eradication, which ultimately results in the recalcitrance and recurrence of biofilm-related implant infection [13,14,15,16].

A biofilm is a complex three-dimensional aggregation of microorganisms, which can be single or multiple species, embedded within the EPS, which has its own internal architecture and nutrient circulation [6,15]. The amount of EPS depends upon the maturity of the biofilm, with one of the most important characteristics of biofilms being the increased tolerance it gives the constituent bacteria to antimicrobial agents due to a combination of advantages offered by its 3D structure as well as the utilisation of conventional resistance pathways; there is decreased antibiotic penetration (EPS acts as a barrier to polar or charged antibiotics and demonstrates antibiotic chelating activity as well as nutrient and chemical diffusion gradients, limiting penetrance through the biofilm), differential bacterial growth rates (ineffective antimicrobial action and tolerance in slowly or non-dividing cells and even the existence of a dormant persistor-state), the induction of resistance mechanisms (such as efflux pumps) and local horizontal gene transfer due to the bacterial cell proximity [15]. Biofilm antibiotic resistance and tolerance mechanisms differing depending on the antimicrobial agent, the bacterial strain and species, the age and developmental phase of the biofilm, and the biofilm growth circumstances [17,18,19,20,21].

## 3. Bacteria in Orthopaedic Implant-Related Infections

The most common bacteria responsible for orthopaedic BRII are Gram-positive *staphylococcal* species namely *Staphylococcus aureus* (*S. aureus*) and coagulase-naegative *staphylococci such as Staphylococcus epidermidis* (*S. epidermidis*), although multiple Gram-positive and negative organisms have been found to be responsible for BRII, and the infection can be caused by a single organism or be polymicrobial [22]. The typical infective species vary by anatomical site: for example, *Cutibacterium acnes* (*C. acnes*) is more prevalent in areas with higher concentration of pilosebaceous follicles of the skin, i.e., the face, neck, shoulder, and back. Therefore, *C.* acnes is most typically found in BRII following shoulder and spine surgery [23]. Acute aggressive implant infections are often caused by more virulent pathogens such as *S. aureus* where more indolent chronic infections are typically caused by commensal bacterial that form part of the skin’s microbiome, such as *S. epidermidis*, where their role in opportunistic biofilm-associated implant infection is well established [24]. In a recent study utilising a murine septic implant model, it was observed that when *S. epidermidis* biofilm was established, it inhibited bone integration at the implant interface and stimulated a pro-inflammatory environment, causing incomplete osseous integration of the implant, but without the aggressive osteolysis, reactive bone formation, or marrow ablation seen with *S. aureus species* [25]. This chronic indolent BRII, often with opportunistic commensal bacteria, is often missed or diagnosed as aseptic loosening due to the lack of overt clinical signs and symptoms as well as negative diagnostic tests including culture-negative samples; it has been reported that up to 42.1% of orthopaedic prosthetic joint infection (PJI) is culture negative [26]. With respect to polymicrobial infection on implant material, a recent study demonstrated the synergistic benefit to micro-organisms in polymicrobial biofilms; in this in vitro study, a combination of *S. aureus* and *Escherichia coli* (*E. coli*) were able to independently establish a biofilm on the surface of implant grade titanium; however, in co-culture, the combined *S. aureus–E. coli* biofilm that formed was significantly more complex and had a higher number of bacteria than that of the simple *S. aureus* or the simple *E. coli* biofilms, demonstrating the mutual benefit in polymicrobial biofilm formation [27].

*S. aureus* and *S. epidermidis* are proficient biofilm-forming bacteria. As well as the immune and pharmacological tolerance conferred by the biofilm, there are also increasing rates of antimicrobial resistance seen in isolate strains causing BRII [28]. Infection caused by multidrug-resistant organisms (MDROs, i.e., those with resistance to more than one antimicrobial drug) results in inadequate or delayed antimicrobial treatment and is associated with poorer outcomes [29,30,31]. In a recent study, there was an attempt to untangle the degree of antimicrobial tolerance/resistance in BRII afforded by either the inherent drug resistance of the isolate strain or the protection offered by biofilm formation; over a 4-year period, clinical bacterial isolates were collected from patients with BRII, and the most common strains were *S. epidermidis* (42%) followed by *S. aureus* (15.6%) [32]. The clinical isolate strains were tested to determine their inherent anti-microbial resistance and also their ability to from biofilms in vitro, which is determined by counting colony-forming units, metabolic activity, and biofilm complexity using scanning electron microscopy, and they found that isolates that were inherently less tolerant to multiple antibiotics were better at forming biofilm in vitro, which likely gave them antimicrobial tolerance in vivo [32].

The combination of innate antimicrobial resistance and the protection conferred by the biofilm structure means that the minimum inhibitory concentration (MIC) of antibiotics required to be available locally is very high [33]. As a result, antibiotics, if given, are usually present in subinhibitory concentrations, which in turn can actively promote biofilm formation. A recent study has demonstrated the effect of subinhibitory concentrations of azithromycin on promoting *Pseudomonas aeruginosa* biofilm formation using species isolated from large joint infections [34]. In another recent study, subinhibitory antibiotic concentrations were demonstrated to increase the number and total protein content of extracellular vesicles (a bacterial communication system that contain a range of cargo molecules including nucleic acids, proteins, lipids, and enzymes and have been observed in several Gram-positive species) in *S. epidermidis*, which cause a modulation in cellular growth rate and cellular division time as well as altering cellular adhesion [35].

Much of our understanding of the microbial behaviour of BRII comes from in vitro studies, and a diversity of approaches have been used [33,36,37,38,39,40,41,42,43,44]. Additionally, clinical, wild-type, bacterial isolates demonstrate phenotypical characteristics that differ significantly from typical laboratory reference strains of the same species; clinical samples tend to be more avid biofilm formers, and bacteria isolated from the disruption of biofilms through sonication form biofilms more readily than organisms obtained through aspiration of joint fluid [11,45]. Ultimately, any in vitro model of orthopaedic BRII is a compromise between creating a realistic model of the in vivo environment versus creating a versatile model that can be reproduced, so any in vitro result must be taken in this context [46]. Table 2 summarises recent publications on different strategies to reduce biofilm formation on orthopaedic implants.

## 4. Novel Implant Surface Technology

Great efforts have been made to devise materials and coatings that prevent or retard bacterial adhesion and hence the formation of biofilms. Bacterial adhesion to surfaces is controlled by physicochemical factors (including surface chemistry, topography, and roughness), bacterial properties (including bacterial hydrophobicity, surface load, and cell size), and environmental parameters (including flow rate, temperature, and pH) [47]. The interaction between bacteria and implant surfaces is balanced between electrostatic and van der Waals forces, and if the bacteria have enough energy to overcome the energetic barrier, then they can become strongly and irreversibly adhered to the material surface [47].

Despite the multifactorial causes that lead to BRII, bacterial adhesion to the implant surface is a common and essential step in all instances. This is an excellent therapeutic target to eradicate biofilm infection before it has even become established and at a stage when the bacteria are still susceptible to conventional antibiotics and host immune attack [48]. The ideal implant surface would be one that minimises bacterial adhesion, inhibits biofilm formation, and confers an effective bactericidal action [49]. Much research has been undertaken in this area, and due to the myriad of possibilities and to compare novel strategies, the different technologies have been classified into passive surface modification, active surface modification, and local carriers and coatings [49].

## 5. Passive Surface Modification

All commonly used orthopaedic implant materials are susceptible to colonisation by biofilm-forming bacteria including ceramics, cobalt–chromium, polyethylene, poly-methyl methacrylate, and titanium alloys [50,51]. On implantation, the implant surface is almost completely and instantaneously covered in a proteinaceous layer (including extracellular matrix proteins and immune protein components), and this process is determined by the wettability and surface chemistry of the implant material [22,52]. Numerous surface factors affecting bacterial adhesion have been investigated including chemical structure, surface roughness, hydrophilicity, Z potential, and surface free energy, but in general, the more inert a surface, the less likely it is to bind bacteria or host conditioning proteins, which then in turn will bind bacteria [52,53,54]. Certain surface characteristics are more attractive to different types of bacteria in different environmental conditions depending on their surface charge and hydrophobicity; however, surfaces that appear least attractive to bacteria tend to exhibit hydrophilic, highly hydrated, and non-charged properties, although there are numerous examples in nature of anti-adhesion super-hydrophobic surfaces [48]. The aim of passive surface modification (PSM) is to reduce bacterial adhesion through altering the implant surface chemistry and/or surface structure modification without local release or surface capture of bactericidal agents [49]. PSM can involve materials already in use or novel materials or coatings.

*Metals*

The tendency of specific bacteria to colonise specific materials may need to be taken into account when choosing implants in specific clinical situations; in a recent in vitro study, *C. acnes* demonstrated greater initial attachment and biofilm formation (in the first 24 h) to polyether ether ketone (PEEK) as compared to cobalt chromium (CC), stainless steel (SS), titanium, and titanium alloy and should be considered in designing shoulder and spine implants, which are regions where *C. acnes* BRII is more common [23]. Smooth implant surfaces appear to discourage the adherence of bacteria, and the converse also appears to be the case; in a recent in vitro study, it was noted that areas of surface imperfections or rougher microtopography harboured the greatest bacterial presence [23,47].

*Ceramics*

Ceramics have been shown to display advantageous physical–chemical surface properties to deter biofilm formation in vitro compared to other implant materials demonstrating reduced bacterial adhesion and slower biofilm development, and there has also been clinical evidence of increased bacterial counts on polyethylene liners compared with ceramic in BRII [55,56,57,58]. In some large cohort studies, it has been demonstrated that after two years post-surgery, there is reduced incidence of infective revision with ceramic bearings compared to other bearing surface combinations, although this delayed effect may not be purely due to the enhanced physical–chemical surface properties but could instead be due to the tendency for bioceramics to undergo little surface degradation compared to metals and polymers, and therefore retain their surface smoothness in the medium to long term [55,59]. More recently, ceramicised metal bearings, such as zirconium oxide, titanium niobium nitride, or titanium nitride, have been developed as a potential solution to prevent metal sensitivity after arthroplasty [60,61,62,63,64]. The ability of ceramicised metals to deter BRII has not been fully established; however, in a recent study, microbial affinity with a titanium-niobium nitride (TiNbN) coating was assessed against common bacterial species that cause BRII; the TiNbN surface had significantly less *S. aureus* and *S. epidermidis* adhered in the two hours of contact compared to CoCrMo, but no difference is found with *Pseudomonas Aeruginosa* or *Candida albicans*; however, there was no significant difference in biofilm formation [65]. Oxinium, oxidised zirconium–niobium alloy, has also been assessed in vitro and showed no significant reduction in adherence compared to common implant materials and was actually significantly more attractive to bacteria than Co-Cr-Mo, which was more hydrophobic [66,67].

*Nanopatterning*

Most novel PSM strategies remain in the preclinical development phase including the modification of the implant surface topographically by the application of a nanopatterning (modifying the surface finish at the nanometer scale) typically on titanium or titanium alloy implants, including the creation of surface nanopores using hydrothermal treatment, the fabrication of nanotube arrays, or surface nanowires, which has demonstrated efficacy in vitro at deterring biofilm formation [68,69,70,71,72]. The chemical modification of implant surfaces by the application of polymer coatings, including hydrophilic polymethacrylic acid, polyethylene oxide, or protein-resistant polyethylene glycol to the surface of titanium implants results in a significant inhibition of bacterial adhesion [49]. In a recent study, both these techniques were combined, a nanopatterned coating mimicking shark skin was subsequently coated with a peptide-based coating and then inoculated with *E. coli* and *S. epidermidis*, thus combining a topographical and chemical PSM anti-microbial surface technology; both surfaces on their own demonstrated efficacy compared to a control smooth surface, but the surface modifications acted synergistically with a dramatic reduction in bacterial cell adhesion and biofilm formation [73].

*Ultraviolet Irradiation of Titanium Dioxide*

PSM strategies described and trialled in vitro include the ultraviolet irradiation of titanium dioxide, which has been shown to increase the wettability of the material surface, and this decreased bacterial adhesion without affecting osteointegration [74], the modification of the crystalline structure of the surface oxide layer to create an anti-bacterial surface [1], and the efficacy of hydrophobic and super hydrophobic surface treatment technologies have also shown to have an anti-bacterial adhesion effect [75].

*Biosurfactants*

Another PSM strategy that is low toxicity and biodegradable is the use of biosurfactants; in one in vitro study, using the biosurfactant lipopeptide, produced by *Bacillus Subtilis ATCC* 19,659, to precondition titanium and stainless steel reduced the adhesion of *E. coli* and *S. aureus* [76]. In a rabbit PJI model, grit-blasted pure titanium implants were coated with cross-linked albumin and compared against an uncoated control and were exposed to *S. epidermidis* before implantation; the cross-linked albumin implants experienced a lower infection rate than the uncoated implants [77].

*Bioactive Glass*

Bioactive glass is being investigated as a potential implant surface coating due to the osteoconductive properties due to the formation of a carbonated hydroxyapatite layer which forms after emersion in aqueous solution, but the adherence of this surface to bacteria is still being investigated [78,79]. BGF18, a bioactive glass containing SiO_2_-Na_2_O-K_2_O-MgO-CaO-P_2_O_5_-coated titanium was assessed against a titanium control and inoculated with *Candida albicans, Pseudomonas aeruginosa*, and *S. epidermidis*; the study found that initial (up to 8 h) bacterial adhesions were reduced with the BGF18 coating; however, interestingly, at 48 h, the biofilm established on the BGF18 surfaces was thicker, and it is postulated that this may be due to glass consumption augmenting biofilm formation; the authors reached the conclusion that BGF18 could potentially prevent infection in situ if the implant is exposed to small bacterial concentrations [79]. In another recent study on BGF18, no clear inhibitor effect on *Candida albicans, Pseudomonas aeruginosa*, and *S. epidermidis* biofilm growth was seen; however, the BGF18 surface did appear to modulate virulence factor gene expression, which is assessed through real-time PCR, although further work is needed to determine the significance of this finding [78].

*Bioactive Molecules*

One concern regarding anti-adhesive coatings is the potential to impair osteoblast adhesion and then osseointegration, leading to early mechanical failure; this has been countered with the inclusion of bioactive molecules such as arginine–glycine–aspartic acid (RGD) peptides and sericin, which can restore or even improve this, and nanopatterned materials have been shown to promote the organisation and proliferation of fibroblasts and osteoblasts at their surface [68,69,71,80,81].

## 6. Active Surface Modification

Active surface modification (ASM) consists of coatings that feature pharmacologically active pre-incorporated bactericidal agents, such as antibiotics, antiseptics, metal ions, non-metal ingredients (e.g., iodine, selenium), organic molecules, and their combinations [49]. ASM may be either contact killing or drug eluting and can be degradable or non-degradable [49]. The bactericidal actions in ASM include inhibiting cell respiration or division, cell wall formation, the bacterial signalling network, as well as inhibition of the transition from free-floating bacteria to aggregates in the biofilm [49]. To consider ASM coatings, we can further split this class into inorganic and organic molecules.

*Inorganic Molecules*

**Figure 5:**
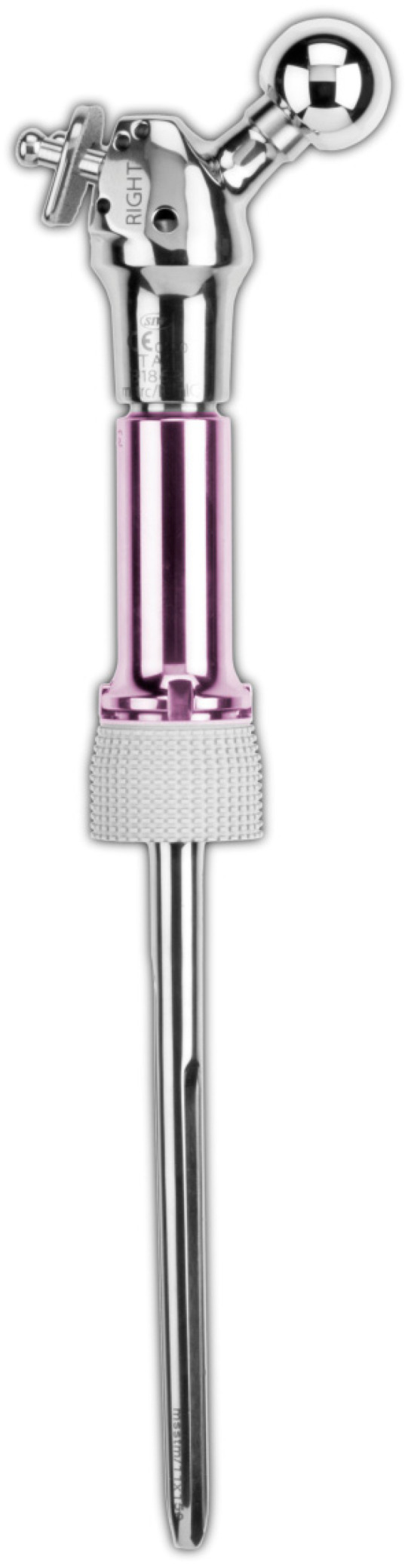
Agulna^®^ silver coating (Stryker, Newbury, UK)—an example of active surface modification.

The most commonly used inorganic metal ion coating is silver, with established products already on the market (Figure 5); the coating functions as dissolved biochemically active silver cations that interfere with bacterial cell membrane permeability, cell metabolism, and create reactive oxygen species, giving it broad-spectrum activity against both Gram-positive and Gram-negative bacteria, fungi, protozoans, and viruses [49,82,83]. However, with a silver coating, there is a risk of host silver toxicity; locally elevated silver concentrations are toxic to osteoblasts, and this may be implicated in osteolysis prosthesis loosening. This theoretical risk should be balanced against the safety and efficacy of silver-coated megaprostheses, which have been demonstrated in clinical studies [84,85,86,87,88,89,90,91,92].

The addition of silver to hydroxyapatite (Ag-HA) coating has been assessed to ameliorate the enhanced bacterial affinity to the hydroxyapatite-coated surfaces demonstrated in both laboratory and clinical studies, especially given the excellent ingrowth and enhanced osteoconductivity that the HA offers [93,94]. Here, 3% silver-HA demonstrated a reduction in the bound methicillin-resistant *S. aureus* and good hydroxyapatite-forming ability, which is a measure of bioactivity, in vitro, and an increase in osteoconductivity in animal models [95,96,97]. In animal studies, the safety profile, with respect to cytotoxicity, has been assessed, and the efficacy of the osseointegration of Ag-HA coatings has also been previously demonstrated [97,98]. In a recent animal study, the synergistic effects of Ag-HA and vancomycin (antibiotic) were demonstrated in a rat methicillin-resistant *S. aureus* infection model where the biofilm that adhered to the Ag-HA disc was thinner and smaller than the plain Ti-disc, but when Ag-HA was combined with vancomycin, the viable counts in vivo were least [99].

Silver nanoparticles (AgNP) coatings have also been applied to CoCr and titanium alloy nanostructured materials and assessed using an American Standard Test Method-E2647-13 (an industry standard to assess the quantity of *Pseudomonas aeruginosa* grown in a drip flow biofilm reactor in low shear and continuous flow); however, in this study, there was no benefit seen with the additional AgNP coating. Instead, it served only to upregulate the *Pseudomonas aeruginosa* virulence factors [100].

Metal ions other than silver have been assessed as anti-microbial coatings including copper (Cu) and zinc (Zn); in a recent paper, these were combined to make a series of Zn-Cu alloys. After mechanical and biocompatibility testing, the Zn-2Cu alloy was further assessed to determine its anti-bacterial activity; the biodegradable Zn-2Cu alloy demonstrated strong anti-microbial activity, preventing bacterial adhesion and biofilm formation against methicillin-resistant *S. aureus* and *S. epidermidis* strains as well as downregulated the expression of genes related to wall synthesis, adhesion, colonisation, biofilm formation, autolysis, and secretion of virulence factors [101]. Similarly, zinc oxide nanomaterials have been demonstrated to prevent bacterial surface adhesion. Li et al. explored the potential mechanisms of action [102]. Direct contact killing; reactive oxygen species (ROS) production; and released zinc ion inactivation were all found to play a role in deterring bacterial adhesion. These toxic effects caused the destruction of bacterial membrane, denaturation of enzyme, and inhibition of cellular respiration and deoxyribonucleic acid replication, producing leakage of the cytoplasmic content and eventual cell apoptosis.

Non-metallic elements, including hydrogen, chlorine, and iodine have been tested in vitro, but questions remain over long-term toxicity; however, in a clinical trial, long-term excellent results have been seen in iodine-coated titanium megaprostheses [49,103]. Other non-metal ions, such as selenium, bound covalently to titanium alloys, have demonstrated efficacy in vitro at reducing the adhesion of *S. epidermidis* but not of osteoblasts [104].

*Organic*

Organic ASMs include antibiotic, coated, or covalently linked (e.g., antibiotic-loaded D-poly-lactate acid/gentamycin-coated intramedullary nails), which are coatings that have been utilised to good effect. However, concerns exist over the efficacy of surface-bound antibiotics, including their limited interaction range relative to their bound position, their effectiveness against only specific bacteria, and the potential for bacterial resistance [49,105,106,107,108,109]. Titanium nanotubes (NT) have been utilised as carriers of bactericidal chemicals coupled with the enhanced osseointegration found with titanium nanotube surfaces [110]. Gentamicin-charged nanotubes can significantly improve anti-bacterial capacity and encourage increased osteoblastic functionality, but again, concerns exist over antibiotic resistance associated with the use of depot antimicrobials [111].

Other organic ASM molecules including chlorhexidine, chloroxylenol, and poly-hexamethylenebiguanide have been shown to be efficacious and may potentially avoid drug resistance [49]. Titanium nanotubules can be loaded with andrographolide, a tradition herbal medicine with known anti-inflammatory properties as well as more recently demonstrated anti-bacterial properties; in one study, the nanopatterning demonstrated biocompatibility, increasing the proliferation of mesenchymal stem cells on the surface as well as limited anti-biofilm activity, but the andrographolide-loaded NT inhibited biofilm formation, which was likely by the elimination of free floating bacteria in suspension, by the local andrographolide release, prior to surface attachment over the time period of the in vitro study [110]. The use of sphingosine, a naturally occurring amino alcohol, was investigated as a surface coating for steel and titanium wires, which significantly reduced bacterial adhesion, demonstrated bacterial colonisation, and significantly reduced biofilm formation on implant surfaces with concentrations greater than 5 μM [112]. Another organic ASM, chitosan, a cationic linear polysaccharide derived from chitin—an abundant biopolymer in insect and crustacean exoskeletons whose mechanism of action is not fully described, but it exhibits anti-bacterial and antifungal activity when coated over titanium alloys—also demonstrates anti-bacterial effects either alone or together with other anti-microbial agents [49,113].

More complex ASM involve multifunctional surface layers combining anti-adhesive and anti-microbial substances and often compounds to enhance tissue integration; one such layer that has shown efficacy in vitro is the anti-adhesive polymer brush coating with anti-microbial peptides (AMP) (naturally occurring substances that have broad anti-microbial action against bacteria, fungi, and viruses, and their mechanism of action includes creating pores in microbial cell membranes causing lysis, and they are less prone to microbial resistance compared to conventional antibiotics) and RGD peptides, which reduce bacterial adhesion and biofilm formation as well as enhance tissue integration [80]. Smart coatings have also been designed to sense the presence of bacteria and then deliver their anti-microbial “payload”, but challenges persist with this technology that need to be overcome before translation into clinical care [48,114]. Qiu et al. reviewed the use of anti-microbial polymers as surface coatings to prevent adhesion, including polypyridine (PPy) derivatives, poly ionic liquids, guanidine-functionalised polymers, conjugated oligomers (COEs), dendritic polyethylene imine (PEI), and anti-microbial peptides. These are generally at preclinical stages of development but hold promise as non-antibiotic ASM agents [115].

## 7. Local Carriers and Coatings

Local carriers or coatings, which may be biodegradable or not, are applied at the time of surgery and can be applied to the implant itself or the peri-implant environment [49]. A key benefit is that they can be used in conjunction with a conventional implant. Antibiotic-eluting PMMA has a long-established clinical practice; however, questions still remain over its use as a carrier, as antibiotic concentrations may not reach the MBEC and sub-therapeutic doses, as described, may encourage of antibiotic tolerance and biofilm formation [49].

*Drug Carriers*

Titanium implants are widely used in trauma and elective orthopaedic practice, but similar to other metals, bacteria can readily form biofilms on titanium implants. Wang et al. describe the creation of an array of titanium dioxide nanotubes (TNTs) on the surface of titanium implants using anodic oxidation. These are proposed to be beneficial to bone ingrowth but could also be used as drug carriers to confer anti-infective properties to the material [116].

*Hydrogel Carriers*

**Figure 6:**
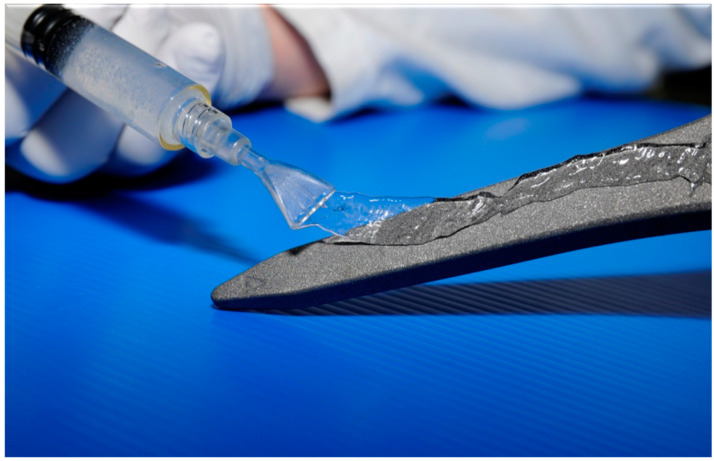
Defensive anti-bacterial coating (DAC; Novagenit, Trento, Italy)—a bioabsorbable, antibiotic-containing hydrogel which is designed to slow or prevent bacterial adhesion and biofilm formation.

A recent strategy has been to design hydrogels with the desired drug elution properties, i.e., high short-term post-implantation antibiotic concentrations above the level that can be achieved by intravenous delivery at a time when the implant is at most risk of colonisation, and which can be loaded with the desired antibiotic regime intra-operatively [117]. One example is a defensive anti-bacterial coating (DAC) that consists of covalently linked hyaluronan and poly-D,L-lactide that undergoes complete hydrolytic degeneration within 72 h, which releases its pre-loaded antibiotics (Figure 6).

In a multicentre randomised controlled trial for the plate osteosynthesis of closed fractures following trauma, it has shown efficacy and a good safety profile [118], and in another recent study, it has tended toward better outcomes when used at second-stage (conversion from antibiotic-loaded spacer) hip revision for prosthetic hip infection [119].

*Biphasic Ceramic Carrier*

Another novel strategy uses a biphasic ceramic-based drug delivery system, a biphasic nanohydroxyapatite/calcium sulphate carrier; a recent paper combining in vitro analysis and computer modelling demonstrated that this void filler not only has the potential to promote bone regeneration, it also demonstrating desirable, controlled, and sustained drug release, which would make it an excellent treatment strategy for bone and joint tuberculosis [14].

## 8. Biofilm Eradication Strategies

Whilst the strategies outlined above exist with the aim of preventing biofilm formation, there remains a need to treat biofilms that have already formed. With established BRII, the only current treatment to ensure complete resolution of the infection is surgical excision of the colonised implant and thorough debridement of the peri-implant environment, as our current therapeutics only serve to suppress, not eradicate, the infection. Novel strategies have been researched to eradicate the biofilm in situ, but so far, there has been limited translation into clinical practice.

Bacteriophages, viruses that specifically infect and kill their bacterial hosts, have been investigated as a treatment strategy due to the rise in anti-microbial resistance, and they have been utilised in some clinical studies demonstrating safety and possible efficacy [120]. Phage-derived lysins, enzymes that cleave peptidoglycan—the main component of the bacterial cell wall, which induces cell lysis—have also been utilised often in combination with bacteriophages and antibiotics [120]. A recent animal study demonstrated the efficacy of a bacteriophage in combination with antibiotics at reducing the bacterial load in a *S. aureus* PJI model; both the bacteriophage and vancomycin antibiotic alone demonstrated modest decreases in the bacterial load, but in combination, they demonstrated a 22.5-fold reduction, which although promising would still be a more appropriate adjunct to current treatments rather than an eradication therapy alone [121]. In another animal study, hydroxypropyl methylcellulose coating was used as a local delivery system for bacteriophages capable of self-multiplication and/or linezolid antibiotics into a murine BRII model where the mice were inoculated with MRSA post-implantation; the dual approach was capable of preventing and treating the implant infection; however, it is likely that the main action was in preventing the biofilm formation rather than biofilm eradication [122]. An injectable hydrogel has also been developed that is capable of encapsulating the bacteriophages against *Pseudomonas aeruginosa* and bringing the active phage to areas of bone infection; a recent study demonstrated that the bacteriophages retain their bacteriolytic activity after encapsulation and release from the hydrogel deliver systems and did not influence the metabolic activity of human mesenchymal stromal cells; the bacteriophage-encapsulating hydrogels were able to show efficacy in reducing bacterial cell counts in a murine BRII model seven days after implantation [123].

The use of sphingosine, a naturally occurring amino alcohol, was previously discussed as a novel implant coating, and the efficacy in solution has also been demonstrated in the eradication of 99.999% of sessile *S. epidermidis* biofilm grown on orthopaedic implant material in vitro, although the authors do note that its effect on host tissue in vivo has not been examined [112].

Another potential biofilm eradication treatment to be trialled in vitro is the use of the aqueous extract from marine sponge-isolated *Enterobacter* strain 84.3, which was selected from 85 bacterial isolates after initial tests suggested it was likely to include some anti-biofilm agent. In the study, the 84.3 extract was able to significantly reduce the biofilm of the *S. epidermidis* and *S. aureus* species as well as being non-toxic to mammalian cells, although the specific anti-biofilm substance or substances remain elusive [28].

The use of conventional antibiotics to inhibit and eradicate *S. aureus* biofilms has been previously assessed, and the biofilm eradication concentration is known to be many factors greater than in free floating cells; however, in a recent in vitro study, this assertion was tested against a single laboratory strain of *S. aureus* where the authors were able to demonstrate biofilm eradication with Rifampicin, whose minimum biofilm inhibitory concentration (MBIC) and minimum biofilm eradication concertation (MBEC) were similar (80 ng/mL), and vancomycin, whose MBIC was 1 μg/mL and MBEC was 6 mg/mL [124]. This paper also underlines the importance of choosing anti-microbial agents such as rifampicin, which disrupts bacterial DNA transcription and whose mechanism of action is appropriate to BRII as opposed to anti-microbials that target cell wall synthesis, which are more effective in rapidly dividing cell lines [124]. Another antibiotic therapy is dalbavancin, a semi-synthetic lipoglycopeptide antibiotic that was assessed using a rat *MRSA*-PJI model, and they found that although treatment for 14 days demonstrated a marked decrease in the number of CFUs, signs of biofilm-induced infection prevailed, and they concluded that further studies should be conducted to evaluate the potential of dalbavancin in treating orthopaedic BRII *MRSA* infections [125]. In another study on dalbavancin, there were achievable concentrations in bone and joint tissue, and the antibiotic showed potent activity against staphylococcal biofilms. Furthermore, compared to vancomycin, dalbavancin was more effective [126].

Another novel treatment strategy is the use of radioimmunotherapy to target planktonic and biofilm state bacteria, and the efficacy has been demonstrated: it had the ability to create specific antibodies to target cell wall teichoic acids that were bacterial cell and biofilm-specifically charged with an alpha-emitter Bismuth-213 to selectively kill *S. aureus* cells in vitro both in planktonic and biofilm states [127].

Another novel strategy is the use of engineered cationic amphipathic peptide, WLBU2, as an irrigation adjunct to be used during the surgical debridement of BRII; WLBU2 has been previously shown to eliminate biofilms and create culture negative implants in 30 min, and in a recent in vitro and in vivo murine infection model, WLBU2 solution was able to markedly reduce *S. aureus* biofilm on implant material [128]. The use of pulsed-lavage irrigation fluid in combination with physiological concentrations of antibiotic agents, vancomycin and flucloxacillin, was demonstrated in vitro to reduce the bacterial load of biofilms adhered to implant materials but did not demonstrate complete eradication [14].

A complex new strategy that has shown exciting early results both in vitro and in vivo animal studies is the utilisation of multiple synergistic anti-microbial mechanisms in combination. A recent paper used titanium oxide nanorod arrays in combination with irradiation of 808 nm near-infrared (NIR) light to eradicate biofilms by combining photothermal therapy, photodynamic therapy, and physical removal of bacteria; this is coupled with the non-toxic TiO2 nanostructures, which promote the proliferation, spreading, and differentiation of osteoblasts in the presence of NIR light irradiation. Ultimately, the technique demonstrated a rapid bactericidal effect that was biosafe [129]. In another recent in vivo study using a rat *MRSA* implant infection model, a hydrophilic and viscous hydrogel of polyvinyl alcohol modified with chitosan, polydopamine, and NO release donor was formed on a red phosphorus nanofilm deposited on a titanium implant (Ti-RP/PCP/RSNO) that was irradiated with NIR, which formed peroxynitrite (in reaction between nitric oxide (NO) and the superoxide free radicals) and eradicated the established *MRSA* biofilm in combination with the hyperthermia and superoxide free radicals created by the NIR irradiation. The hydrogel also eradicated the immunoreaction due to the creation of NO, which caused the M1 polarisation of local macrophages in vivo and was responsible for the development of an anti-inflammatory and tissue regenerative environment, leading to excellent bone formation post-irradiation [130]. In another study on the efficacy of non-contact induction heating (NIH) as a treatment in prosthetic joint infection, the synergistic action of antibiotics and N-acetylcysteine (NAC) was also assessed, and the results demonstrated the efficacy of NIH to reduce the bacterial load of *S. aureus* biofilms but also that in combination at 60 °C with clinically relevant concentrations of vancomycin, rifampicin (antibiotics), and NAC, total eradication of the biofilm was observed [131].

Photodynamic therapy (PDT) potentially has a role in the prevention and disruption of biofilms. In PDT, a photosensitising agent (for instance, methylene blue) is used with a specialised light source to create a toxic effect on biofilms. Briggs et al. demonstrated a toxic effect of PDT using a laser light source and methylene blue on biofilms in vitro [132]. Bapat et al. have demonstrated a similar effect on fungal biofilms with inhibition of the formation and disruption of mature biofilms, both with and without the addition of photosensitising compounds [133].

## 9. Conclusions

The infection of orthopaedic implants has a significant socioeconomic impact. To prevent bacterial adhesion to orthopaedic implants and subsequent infection, it is necessary to start with adequate preoperative care, appropriate surgical techniques, and good postoperative care. The ideal implant material or surface coating would be able to perform its function whilst being immune to bacterial colonisation and super compatible with the host tissue, i.e., promoting osseointegration and inhibiting infection. Numerous strategies, namely passive surface modifications, active surface modifications, and local carriers, have been developed and are being interrogated to determine their potential clinical efficacy. To treat BRII, a swift reliable diagnosis must be made, which should ideally determine if an infection is present, if a biofilm has been formed, and what microorganism or microorganisms are responsible, and active research continues in this endeavour. Finally, after a BRII has been diagnosed, at present, the only treatment strategy to eradicate the infection is implant removal, but novel strategies, using vastly different technologies, are offering potential solutions to purge the implant of infection whilst remaining in situ.

## Figures and Tables

**Table 1:** Prevention of orthopaedic implant-related infection.

Prevention of Orthopaedic Implant-Related Infection
Optimisation of Preoperative Medical Conditions
Systemic prophylaxis in a 24-h regimen
Good clinical and logistic practice in operating rooms
Optimal air conditions
Shorter surgical interventions
Anti-infective coatings and implants
Optimal postoperative treatment

**Table 2:** Summary of recent publications on different strategies to reduce biofilm formation on orthopaedic implants.

Summary of Recent Publications on Different Strategies to Reduce Biofilm Formation on Orthopaedic Implants.
Local administration of nanotube-based antibiotics could be a good future strategy to combat infections associated with orthopaedic implants.
Combination of silver-containing hydroxyapatite coating and vancomycin reduces the formation of MRSA biofilms and, therefore, could be useful to prevent and treat periprosthetic joint infections.
Peptide-coated surface with micropatterned topography can reduce adhesion and subsequent biofilm formation on orthopedic implants.
An effective strategy to eliminate biofilms is to use phages or lysins in combination with antibiotics.
Bacteriophage-encapsulating hydrogels may be useful for treating local bone infections.
Implants with uniform surfaces and minimal imperfections can reduce the ability of bacteria to adhere to them.
Sponge-associated *Enterobacter* 84.3 is a source of substances capable of eradicating staphylococcal biofilm.
The lipopeptide produced by *Bacillus subtilis* ATCC 19,659 may be of great interest in the future to avoid infections related to orthopedic implants.
MRSA = Methicillin-resistant *Staphylococcus Aureus*

## References

[1] Filipovic U., Dahmane R.G., Ghannouchi S., Zore A., Bohinc K. (2020). Bacterial adhesion on orthopedic implants. Adv. Colloid Interface Sci..

[2] Moriarty T.F., Schlegel U., Perren S., Richards R.G. (2010). Infection in fracture fixation: Can we influence infection rates through implant design?. J. Mater. Sci. Mater. Med..

[3] Kapadia B.H., Berg R.A., Daley J.A., Fritz J., Bhave A., Mont M.A. (2016). Periprosthetic joint infection. Lancet.

[4] Vastag B. (2004). Knee replacement underused, says panel: Useful option when nonsurgical therapies fail. JAMA.

[5] Lamagni T. (2014). Epidemiology and burden of prosthetic joint infections. J. Antimicrob. Chemother..

[6] Saeed K., McLaren A.C., Schwarz E.M., Antoci V., Arnold W.V., Chen A.F., Clauss M., Esteban J., Gant V., Hendershot E. (2019). The 2018 International Consensus Meeting on Musculoskeletal Infection: Summary from the Biofilm Workgroup and consensus on Biofilm related Musculoskeletal Infections. J. Orthop. Res..

[7] Jamal M., Ahmad W., Andleeb S., Jalil F., Imran M., Nawaz M.A., Hussain T., Ali M., Rafiq M., Kamil M.A. (2019). Bacterial biofilm and associated infections. J. Chin. Med. Assoc..

[8] Gristina A.G. (1987). Biomaterial-centered infection: Microbial adhesion versus tissue integration. Science.

[9] McConda D.B., Karnes J.M., Hamza T., Lindsey B.A. (2016). A novel co-culture model of murine K12 osteosarcoma cells and *S. aureus* on common orthopedic implant materials: ‘The race to the surface’ studied in vitro. Biofouling.

[10] Donlan R.M., Costerton J.W. (2002). Biofilms: Survival mechanisms of clinically relevant microorganisms. Clin. Microbiol. Rev..

[11] Costerton J.W. (1995). Overview of microbial biofilms. J. Ind. Microbiol..

[12] Flemming H.C., Wingender J., Szewzyk U., Steinberg P., Rice S.A., Kjelleberg S. (2016). Biofilms: An emergent form of bacterial life. Nat. Rev. Microbiol..

[13] Mottola C., Matias C.S., Mendes J.J., Melo-Cristino J., Tavares L., Cavaco-Silva P., Oliveira M. (2016). Susceptibility patterns of *Staphylococcus aureus* biofilms in diabetic foot infections. BMC Microbiol..

[14] Poilvache H., Ruiz-Sorribas A., Sakoulas G., Rodriguez-Villalobos H., Cornu O., Van Bambeke F. (2020). Synergistic effects of pulsed lavage and antimicrobial therapy against *Staphylococcus aureus* biofilms in an in-vitro model. Front. Med..

[15] Macia M.D., Rojo-Molinero E., Oliver A. (2014). Antimicrobial susceptibility testing in biofilm-growing bacteria. Clin. Microbiol. Infect..

[16] Nishimura S., Tsurumoto T., Yonekura A., Adachi K., Shindo H. (2006). Antimicrobial susceptibility of *Staphylococcus aureus* and *Staphylococcus epidermidis* biofilms isolated from infected total hip arthroplasty cases. J. Orthop. Sci.

[17] Ibrahim E.H., Sherman G., Ward S., Fraser V.J., Kollef M.H. (2000). The influence of inadequate antimicrobial treatment of bloodstream infections on patient outcomes in the ICU setting. Chest.

[18] Ito A., Taniuchi A., May T., Kawata K., Okabe S. (2009). Increased antibiotic resistance of *Escherichia coli* in mature biofilms. Appl. Environ. Microbiol..

[19] Alhede M., Kragh K.N., Qvortrup K., Allesen-Holm M., van Gennip M., Christensen L.D., Jensen P.O., Nielsen A.K., Parsek M., Wozniak D. (2011). Phenotypes of non-attached *Pseudomonas aeruginosa* aggregates resemble surface attached biofilm. PLoS ONE.

[20] Bowler L.L., Zhanel G.G., Ball T.B., Saward L.L. (2012). Mature Pseudomonas aeruginosa biofilms prevail compared to young biofilms in the presence of ceftazidime. Antimicrob. Agents Chemother..

[21] Haaber J., Cohn M.T., Frees D., Andersen T.J., Ingmer H. (2012). Planktonic aggregates of *Staphylococcus aureus* protect against common antibiotics. PLoS ONE.

[22] Arciola C.R., Campoccia D., Montanaro L. (2018). Implant infections: Adhesion, biofilm formation and immune evasion. Nat. Rev. Microbiol..

[23] Garcia D., Mayfield C.K., Leong J., Deckey D.G., Zega A., Glasser J., Daniels A.H., Eberson C., Green A., Born C. (2020). Early adherence and biofilm formation of *Cutibacterium acnes* (formerly *Propionibacterium acnes*) on spinal implant materials. Spine J..

[24] Achermann Y., Goldstein E.J., Coenye T., Shirtliff M.E. (2014). Propionibacterium acnes: From commensal to opportunistic biofilm-associated implant pathogen. Clin. Microbiol. Rev..

[25] Tomizawa T., Ishikawa M., Bello-Irizarry S.N., de Mesy Bentley K.L., Ito H., Kates S.L., Daiss J.L., Beck C., Matsuda S., Schwarz E.M. (2020). Biofilm producing *Staphylococcus epidermidis* (RP62A strain) inhibits osseous integration without osteolysis and histopathology in a murine septic implant model. J. Orthop. Res..

[26] Yoon H.K., Cho S.H., Lee D.Y., Kang B.H., Lee S.H., Moon D.G., Kim D.H., Nam D.C., Hwang S.C. (2017). A review of the literature on culture-negative periprosthetic joint infection: Epidemiology, diagnosis and treatment. Knee Surg. Relat. Res..

[27] Dong J., Wang B., Xiang B., Yang J., Gong Z., Wang Z., Huang Y., Chen L. (2020). Research on the effect of TiO_2_ nanotubes coated by gallium nitrate on *Staphylococcus aureus*-*Escherichia coli* biofilm formation. J. Clin. Lab. Anal..

[28] Nunes S.O., Rosa H.D.S., Canellas A.L.B., Romanos M.T.V., Dos Santos K.R.N., Muricy G., Oelemann W.M.R., Laport M.P. (2020). High reduction of staphylococcal biofilm by aqueous extract from marine sponge-isolated *Enterobacter* sp.. Res. Microbiol..

[29] Anderson D.J., Engemann J.J., Harrell L.J., Carmeli Y., Reller L.B., Kaye K.S. (2006). Predictors of mortality in patients with bloodstream infection due to ceftazidime-resistant Klebsiella pneumoniae. Antimicrob. Agents Chemother..

[30] Cosgrove S.E., Sakoulas G., Perencevich E.N., Schwaber M.J., Karchmer A.W., Carmeli Y. (2003). Comparison of mortality associated with methicillin-resistant and methicillin-susceptible *Staphylococcus aureus* bacteremia: A meta-analysis. Clin. Infect. Dis..

[31] Roberts R.R., Hota B., Ahmad I., Scott R.D., Foster S.D., Abbasi F., Schabowski S., Kampe L.M., Ciavarella G.G., Supino M. (2009). Hospital and societal costs of antimicrobial-resistant infections in a Chicago teaching hospital: Implications for antibiotic stewardship. Clin. Infect. Dis..

[32] Coraça-Huber D.C., Kreidl L., Steixner S., Hinz M., Dammerer D., Fille M. (2020). Identification and morphological characterization of biofilms formed by strains causing infection in orthopedic implants. Pathogens.

[33] Stewart P.S. (2015). Antimicrobial tolerance in biofilms. Microbiol. Spectr..

[34] Babushkina I.V., Ulyanov V.Y., Mamonova I.A., Shpinyak S.P. (2020). The effect of azithromycin on biofilms formation by pathogens of implant-associated infection in large joints. Bull. Exp. Biol. Med..

[35] Zaborowska M., Taulé Flores C., Vazirisani F., Shah F.A., Thomsen P., Trobos M. (2020). Extracellular vesicles influence the growth and adhesion of staphylococcus epidermidis under antimicrobial selective pressure. Front. Microbiol..

[36] Hall C.W., Mah T.F. (2017). Molecular mechanisms of biofilm-based antibiotic resistance and tolerance in pathogenic bacteria. FEMS Microbiol. Rev..

[37] Ceri H., Olson M.E., Stremick C., Read R.R., Morck D., Buret A. (1999). The Calgary biofilm device: New technology for rapid determination of antibiotic susceptibilities of bacterial biofilms. J. Clin. Microbiol..

[38] Hall-Stoodley L., Rayner J.C., Stoodley P., Lappin-Scott H.M., Edwards C. (1999). Establishment of experimental biofilms using the modified robbins device and flow cells. Environmental Monitoring of Bacteria.

[39] Merritt J.H., Kadouri D.E., O’Toole G.A. (2005). Growing and analyzing static biofilms. Curr. Protoc. Microbiol..

[40] Sternberg C., Tolker-Nielsen T. (2006). Growing and analyzing biofilms in flow cells. Curr. Protoc. Microbiol..

[41] O’Toole G.A. (2011). Microtiter dish biofilm formation assay. J. Vis. Exp..

[42] Peterson S.B., Irie Y., Borlee B.R., Murakami K., Harrison J.J., Colvin K.M., Parsek M.P., Bjarnsholt T., Jensen P.O., Moser C., Høiby N. (2011). Different methods for culturing biofilms in vitro. Biofilm Infections.

[43] Rumbaugh K.P., Carty N.L., Bjarnsholt T., Jensen P.O., Moser C., Høiby N. (2011). In vivo models of biofilm infection. Biofilm Infections.

[44] Lebeaux D., Chauhan A., Rendueles O., Beloin C. (2013). From in vitro to in vivo models of bacterial biofilm-related infections. Pathogens.

[45] Babushkina I.V., Bondarenko A.S., Ulyanov V.Y., Mamonova I.A. (2020). Biofilm formation by gram-negative bacteria during implant-associated infection. Bull. Exp. Biol. Med..

[46] Malone M., Goeres D.M., Gosbell I., Vickery K., Jensen S., Stoodley P. (2017). Approaches to biofilm-associated infections: The need for standardized and relevant biofilm methods for clinical applications. Expert Rev. Anti-Infect. Ther..

[47] Fink R., Oder M., Rangus D., Raspor P., Bohinc K. (2015). Microbial adhesion capacity. Influence of shear and temperature stress. Int. J. Environ. Health Res..

[48] Gallo J., Holinka M., Moucha C.S. (2014). Antibacterial surface treatment for orthopaedic implants. Int. J. Mol. Sci..

[49] Romano C.L., Scarponi S., Gallazzi E., Romano D., Drago L. (2015). Antibacterial coating of implants in orthopaedics and trauma: A classification proposal in an evolving panorama. J. Orthop. Surg. Res..

[50] Gbejuade H.O., Lovering A.M., Webb J.C. (2015). The role of microbial biofilms in prosthetic joint infections. Acta Orthop..

[51] Nana A., Nelson S.B., McLaren A., Chen A.F. (2016). What’s new in musculoskeletal infection: Update on biofilms. J. Bone Jt. Surg. Am..

[52] Campoccia D., Montanaro L., Arciola C.R. (2013). A review of the biomaterials technologies for infection-resistant surfaces. Biomaterials.

[53] Koseki H., Yonekura A., Shida T., Yoda I., Horiuchi H., Morinaga Y., Yanagihara K., Sakoda H., Osaki M., Tomita M. (2014). Early staphylococcal biofilm formation on solid orthopaedic implant materials: In vitro study. PLoS ONE.

[54] Roberts A.E., Kragh K.N., Bjarnsholt T., Diggle S.P. (2015). The limitations of in vitro experimentation in understanding biofilms and chronic infection. J. Mol. Biol..

[55] Sorrentino R., Cochis A., Azzimonti B., Caravaca C., Chevalier J., Kuntz M., Porporati A.A., Streicher R.M., Rimondini L. (2018). Reduced bacterial adhesion on ceramics used for arthroplasty applications. J. Eur. Ceram. Soc..

[56] Hexter A.T., Hislop S.M., Blunn G.W., Liddle A.D. (2018). The effect of bearing surface on risk of periprosthetic joint infection in total hip arthroplasty. Bone Jt. J..

[57] Lass R., Giurea A., Kubista B., Hirschl A.M., Graninger W., Presterl E., Windhager R., Holinka J. (2014). Bacterial adherence to different components of total hip prosthesis in patients with prosthetic joint infection. Int. Orthop..

[58] Pitto R.P., Sedel L. (2016). Periprosthetic joint infection in hip arthroplasty: Is there an association between infection and bearing surface type?. Clin. Orthop. Relat. Res..

[59] Lenguerrand E., Whitehouse M.R., Beswick A.D., Kunutsor S.K., Burston B., Porter M., Blom A.W. (2018). Risk factors associated with revision for prosthetic joint infection after hip replacement: A prospective observational cohort study. Lancet Infect. Dis..

[60] Lachiewicz P.F., Watters T.S., Jacobs J.J. (2016). Metal hypersensitivity and total knee arthroplasty. J. Am. Acad. Orthop. Surg..

[61] Innocenti M., Vieri B., Melani T., Paoli P., Carulli C. (2017). Metal hypersensitivity after knee arthroplasty: Fact or fiction?. Acta Biomed..

[62] Faschingbauer M., Renner L., Boettner F. (2017). Allergy in total knee replacement. Does it exist?. HSS J..

[63] Van Hove R.P., Sierevelt I.N., van Royen B.J., Nolte P.A. (2015). Titanium-nitride coating of orthopaedic implants: A review of the literature. BioMed Res. Int..

[64] Thienpont E. (2015). Titanium niobium nitride knee implants are not inferior to chrome cobalt components for primary total knee arthroplasty. Arch. Orthop. Trauma Surg..

[65] Bidossi A., Bottagisio M., De Grandi R., De Vecchi E. (2020). Ability of adhesion and biofilm formation of pathogens of periprosthetic joint infections on titanium-niobium nitride (TiNbN) ceramic coatings. J. Orthop. Surg. Res..

[66] Yoda I., Koseki H., Tomita M., Shida T., Horiuchi H., Sakoda H., Osaki M. (2014). Effect of surface roughness of biomaterials on Staphylococcus epidermidis adhesion. BMC Microbiol..

[67] Shida T., Koseki H., Yoda I., Horiuchi H., Sakoda H., Osaki M. (2013). Adherence ability of Staphylococcus epidermidis on prosthetic biomaterials: An in vitro study. Int. J. Nanomed..

[68] Cao Y., Su B., Chinnaraj S., Jana S., Bowen L., Charlton S., Duan P., Jakubovics N.S., Chen J. (2018). Nanostructured titanium surfaces exhibit recalcitrance towards *Staphylococcus epidermidis* biofilm formation. Sci. Rep..

[69] Bhadra C.M., Truong V.K., Pham V.T., Al Kobaisi M., Seniutinas G., Wang J.Y., Juodkazis S., Crawford R.J., Ivanova E.P. (2015). Antibacterial titanium nano-patterned arrays inspired by dragonfly wings. Sci. Rep..

[70] Tang P., Zhang W., Wang Y., Zhang B., Wang H., Lin C., Zhang L. (2011). Effect of superhydrophobic surface of titanium on staphylococcus aureus adhesion. J. Nanomater..

[71] Valdez-Salas B., Beltran-Partida E., Castillo-Uribe S., Curiel-Alvarez M., Zlatev R., Stoytcheva M., Montero-Alpirez G., Vargas-Osuna L. (2017). In vitro assessment of early bacterial activity on micro/nanostructured Ti6Al4V surfaces. Molecules.

[72] Singh A.V., Vyas V., Patil R., Sharma V., Scopelliti P.E., Bongiorno G., Podesta A., Lenardi C., Gade W.N., Milani P. (2011). Quantitative characterization of the influence of the nanoscale morphology of nanostructured surfaces on bacterial adhesion and biofilm formation. PLoS ONE.

[73] Dolid A., Gomes L.C., Mergulhão F.J., Reches M. (2020). Combining chemistry and topography to fight biofilm formation: Fabrication of micropatterned surfaces with a peptide-based coating. Colloids Surf. B Biointerfaces.

[74] Gallardo-Moreno A.M., Pacha-Olivenza M.A., Saldana L., Perez-Giraldo C., Bruque J.M., Vilaboa N., Gonzalez-Martin M.L. (2009). In vitro biocompatibility and bacterial adhesion of physico-chemically modified Ti6Al4V surface by means of UV irradiation. Acta Biomater..

[75] Zhu H., Guo Z., Liu W. (2014). Adhesion behaviors on superhydrophobic surfaces. Chem. Commun..

[76] Pires M.E.E., Parreira A.G., Silva T.N.L., Colares H.C., da Silva J.A., Teixeira de Magalhães J., Sobreira Galdino A., Bonoto Gonçalves D.M., Granjeiro J.M., Granjeiro P.A. (2020). Recent patents on impact of lipopeptide on the biofilm formation onto titanium and stainless steel surfaces. Recent Pat. Biotechnol..

[77] An Y.H., Bradley J., Powers D.L., Friedman R.J. (1997). The prevention of prosthetic infection using a cross-linked albumin coating in a rabbit model. J. Bone Jt. Surg. Br..

[78] Oliveira V.D.C., Souza M.T., Zanotto E.D., Watanabe E., Coraça-Huber D. (2020). Biofilm formation and expression of virulence genes of microorganisms grown in contact with a new bioactive glass. Pathogens.

[79] Marques D.A., Oliveira V.C., Souza M.T., Zanotto E.D., Mardegan Issa J.P., Watanabe E. (2020). Biomaterials for orthopedics: Anti-biofilm activity of a new bioactive glass coating on titanium implants. Biofouling.

[80] Muszanska A.K., Rochford E.T., Gruszka A., Bastian A.A., Busscher H.J., Norde W., van der Mei H.C., Herrmann A. (2014). Antiadhesive polymer brush coating functionalized with antimicrobial and RGD peptides to reduce biofilm formation and enhance tissue integration. Biomacromolecules.

[81] Oh S., Moon K.S., Lee S.H. (2013). Effect of RGD peptide-coated TiO2nanotubes on the attachment, proliferation, and functionality of bone-related cells. J. Nanomater..

[82] Brennan S.A., Ní Fhoghlú C., Devitt B.M., O’Mahony F.J., Brabazon D., Walsh A. (2015). Silver nanoparticles and their orthopaedic applications. Bone Jt. J..

[83] Chaloupka K., Malam Y., Seifalian A.M. (2010). Nanosilver as a new generation of nanoproduct in biomedical applications. Trends Biotechnol..

[84] Hussmann B., Johann I., Kauther M.D., Landgraeber S., Jäger M., Lendemans S. (2013). Measurement of the silver ion concentration in wound fluids after implantation of silver-coated megaprostheses: Correlation with the clinical outcome. BioMed Res. Int..

[85] Fielding G.A., Roy M., Bandyopadhyay A., Bose S. (2012). Antibacterial and biological characteristics of silver containing and strontium doped plasma sprayed hydroxyapatite coatings. Acta Biomater..

[86] Albers C.E., Hofstetter W., Siebenrock K.A., Landmann R., Klenke F.M. (2013). In vitro cytotoxicity of silver nanoparticles on osteoblasts and osteoclasts at antibacterial concentrations. Nanotoxicology.

[87] Brutel de la Riviere A., Dossche K.M., Birnbaum D.E., Hacker R. (2000). First clinical experience with a mechanical valve with silver coating. J. Heart Valve Dis..

[88] Tweden K.S., Cameron J.D., Razzouk A.J., Holmberg W.R., Kelly S.J. (1997). Biocompatibility of silver-modified polyester for antimicrobial protection of prosthetic valves. J. Heart Valve Dis..

[89] Wan A.T., Conyers R.A., Coombs C.J., Masterton J.P. (1991). Determination of silver in blood, urine, and tissues of volunteers and burn patients. Clin. Chem..

[90] Wafa H., Grimer R.J., Reddy K., Jeys L., Abudu A., Carter S.R., Tillman R.M. (2015). Retrospective evaluation of the incidence of early periprosthetic infection with silver-treated endoprostheses in high-risk patients: Case-control study. Bone Jt. J..

[91] Hardes J., von Eiff C., Streitbuerger A., Balke M., Budny T., Henrichs M.P., Hauschild G., Ahrens H. (2010). Reduction of periprosthetic infection with silver-coated megaprostheses in patients with bone sarcoma. J. Surg. Oncol..

[92] Schmidt-Braekling T., Streitbuerger A., Gosheger G., Boettner F., Nottrott M., Ahrens H., Dieckmann R., Guder W., Andreou D., Hauschild G. (2017). Silver-coated megaprostheses: Review of the literature. Eur. J. Orthop. Surg. Traumatol..

[93] Suzuki T., Fujibayashi S., Nakagawa Y., Noda I., Nakamura T. (2006). Ability of zirconia double coated with titanium and hydroxyapatite to bond to bone under load-bearing conditions. Biomaterials.

[94] Lazarinis S., Mäkelä K.T., Eskelinen A., Havelin L., Hallan G., Overgaard S., Pedersen A.B., Kärrholm J., Hailer N.P. (2017). Does hydroxyapatite coating of uncemented cups improve long-term survival? An analysis of 28,605 primary total hip arthroplasty procedures from the Nordic Arthroplasty Register Association (NARA). Osteoarthr. Cartil..

[95] Shimazaki T., Miyamoto H., Ando Y., Noda I., Yonekura Y., Kawano S., Miyazaki M., Mawatari M., Hotokebuchi T. (2010). In vivo antibacterial and silver-releasing properties of novel thermal sprayed silver-containing hydroxyapatite coating. J Biomed. Mater. Res. B Appl. Biomater..

[96] Yonekura Y., Miyamoto H., Shimazaki T., Ando Y., Noda I., Mawatari M., Hotokebuchi T. (2011). Osteoconductivity of thermal-sprayed silver-containing hydroxyapatite coating in the rat tibia. J. Bone Jt. Surg. Br..

[97] Noda I., Miyaji F., Ando Y., Miyamoto H., Shimazaki T., Yonekura Y., Miyazaki M., Mawatari M., Hotokebuchi T. (2009). Development of novel thermal sprayed antibacterial coating and evaluation of release properties of silver ions. J. Biomed. Mater. Res. B Appl. Biomater..

[98] Tsukamoto M., Miyamoto H., Ando Y., Noda I., Eto S., Akiyama T., Yonekura Y., Sonohata M., Mawatari M. (2014). Acute and subacute toxicity in vivo of thermal-sprayed silver containing hydroxyapatite coating in rat tibia. BioMed Res. Int..

[99] Hashimoto A., Miyamoto H., Kobatake T., Nakashima T., Shobuike T., Ueno M., Murakami T., Noda I., Sonohata M., Mawatari M. (2020). The combination of silver-containing hydroxyapatite coating and vancomycin has a synergistic antibacterial effect on methicillin-resistant Staphylococcus aureus biofilm formation. Bone Jt. Res..

[100] Leonetti S., Tuvo B., Campanella B., Legnaioli S., Onor M., Bramanti E., Totaro M., Baggiani A., Giorgi S., Privitera G.P. (2020). Evaluation of microbial adhesion and biofilm formation on nano-structured and nano-coated ortho-prosthetic materials by a dynamic model. Int. J. Environ. Res. Public Health.

[101] Qu X., Yang H., Jia B., Yu Z., Zheng Y., Dai K. (2010). Biodegradable Zn–Cu alloys show antibacterial activity against MRSA bone infection by inhibiting pathogen adhesion and biofilm formation. Acta Biomater..

[102] Li Y., Liao C., Tjong S.C. (2020). Recent advances in zinc oxide nanostructures with antimicrobial activities. Int. J. Mol. Sci..

[103] Shirai T., Tsuchiya H., Nishida H., Yamamoto N., Watanabe K., Nakase J., Terauchi R., Arai Y., Fujiwara H., Kubo T. (2014). Antimicrobial megaprostheses supported with iodine. J. Biomater. Appl..

[104] Holinka J., Pilz M., Kubista B., Presterl E., Windhager R. (2013). Effects of selenium coating of orthopaedic implant surfaces on bacterial adherence and osteoblastic cell growth. Bone Jt. J..

[105] Campoccia D., Montanaro L., Speziale P., Arciola C.R. (2010). Antibiotic-loaded biomaterials and the risks for the spread of antibiotic resistance following their prophylactic and therapeutic clinical use. Biomaterials.

[106] Lee D.W., Yun Y.P., Park K., Kim S.E. (2012). Gentamicin and bone morphogenic protein-2 (BMP-2)-delivering heparinized-titanium implant with enhanced antibacterial activity and osteointegration. Bone.

[107] Diefenbeck M., Schrader C., Gras F., Muckley T., Schmidt J., Zankovych S., Bossert J., Jandt K.D.M., Volpel A., Sigusch B.W. (2016). Gentamicin coating of plasma chemical oxidized titanium alloy prevents implant-related osteomyelitis in rats. Biomaterials.

[108] Fuchs T., Stange R., Schmidmaier G., Raschke M.J. (2011). The use of gentamicin-coated nails in the tibia: Preliminary results of a prospective study. Arch. Orthop. Trauma Surg..

[109] Pinto D., Manjunatha K., Savur A.D., Ahmed N.R., Mallya S., Ramya V. (2019). Comparative study of the efficacy of gentamicin-coated intramedullary interlocking nail versus regular intramedullary interlocking nail in Gustilo type I and II open tibia fractures. Chin. J. Traumatol..

[110] Feng E., Shen K., Lin F., Lin W., Zhang T., Zhang Y., Lin F., Yang Y., Lin C. (2020). Improved osteogenic activity and inhibited bacterial biofilm formation on andrographolide-loaded titania nanotubes. Ann. Transl. Med..

[111] Popat K.C., Eltgroth M., Latempa T.J., Grimes C.A., Desai T.A. (2007). Decreased Staphylococcus epidermis adhesion and increased osteoblast functionality on antibiotic-loaded titania nanotubes. Biomaterials.

[112] Beck S., Sehl C., Voortmann S., Verhasselt H.L., Edwards M.J., Buer J., Hasenberg M., Gulbins E., Becker K.A. (2020). Sphingosine is able to prevent and eliminate *Staphylococcus epidermidis* biofilm formation on different orthopedic implant materials in vitro. J. Mol. Med..

[113] Raphel J., Karlsson J., Galli S., Wennerberg A., Lindsay C., Haugh M.G., Pajarinen J., Goodman S.B., Jimbo R., Andersson M. (2016). Engineered protein coatings to improve the osseointegration of dental and orthopaedic implants. Biomaterials.

[114] Yu Q., Cho J., Shivapooja P., Ista L.K., Lopez G.P. (2013). Nanopatterned smart polymer surfaces for controlled attachment, killing, and release of bacteria. ACS Appl. Mater. Interfaces.

[115] Qiu H., Si Z., Luo Y., Feng P., Wu X., Hou W., Zhu Y., Chan-Park M.B., Xu L., Huang D. (2020). The mechanisms and the applications of antibacterial polymers in surface modification on medical devices. Front. Bioeng. Biotechnol..

[116] Wang K., Jin H., Song Q., Huo J., Zhang J., Li P. (2021). Titanium dioxide nanotubes as drug carriers for infection control and osteogenesis of bone implants. Drug Deliv. Transl. Res..

[117] Pitarresi G., Palumbo F.S., Calascibetta F., Fiorica C., Di Stefano M., Giammona G. (2013). Medicated hydrogels of hyaluronic acid derivatives for use in orthopedic field. Int. J. Pharm..

[118] Malizos K., Blauth M., Danita A., Capuano N., Mezzoprete R., Logoluso N., Drago L., Romano C.L. (2017). Fast-resorbable antibiotic-loaded hydrogel coating to reduce post-surgical infection after internal osteosynthesis: A multicenter randomized controlled trial. J. Orthop. Traumatol..

[119] Zagra L., Gallazzi E., Romano D., Scarponi S., Romano C. (2019). Two-stage cementless hip revision for peri-prosthetic infection with an antibacterial hydrogel coating: Results of a comparative series. Int. Orthop..

[120] Łusiak-Szelachowska M., Weber-Dąbrowska B., Górski A. (2020). Bacteriophages and lysins in biofilm control. Virol. Sin..

[121] Morris J.L., Letson H.L., Elliott L., Grant A.L., Wilkinson M., Hazratwala K., McEwen P. (2019). Evaluation of bacteriophage as an adjunct therapy for treatment of peri-prosthetic joint infection caused by *Staphylococcus aureus*. PLoS ONE.

[122] Kaur S., Harjai K., Chhibber S. (2016). In vivo ssessment of Phage and linezolid based implant coatings for treatment of methicillin resistant *S. aureus* (MRSA) mediated orthopaedic device related infections. PLoS ONE.

[123] Wroe J.A., Johnson C.T., García A.J. (2020). Bacteriophage delivering hydrogels reduce biofilm formation in vitro and infection in vivo. J. Biomed. Mater. Res. A.

[124] Douthit C., Gudenkauf B., Hamood A., Mudaliar N., Caroom C., Jenkins M. (2020). Effects of powdered rifampin and vancomycin solutions on biofilm production of *Staphylococcus aureus* on orthopedic implants. J. Clin. Orthop. Trauma.

[125] Silva V., Antão H.S., Guimarães J., Prada J., Pires I., Martins A., Maltez L., Pereira J.E., Capelo J.L., Igrejas G. (2020). Efficacy of dalbavancin against MRSA biofilms in a rat model of orthopaedic implant-associated infection. J. Antimicrob. Chemother..

[126] Di Pilato V., Ceccherini F., Sennati S., D’Agostino F., Arena F., D’Atanasio N., Di Giorgio F.P., Tongiani S., Pallecchi L., Rossolini G.M. (2020). In vitro time-kill kinetics of dalbavancin against *Staphylococcus* spp. biofilms over prolonged exposure times. Diagn. Microbiol. Infect. Dis..

[127] Van Dijk B., Allen K.J.H., Helal M., Vogely H.C., Lam M.G.E.H., de Klerk J.M.H., Weinans H., van der Wal B.C.H., Dadachova E. (2020). Radioimmunotherapy of methicillin-resistant Staphylococcus aureus in planktonic state and biofilms. PLoS ONE.

[128] Mandell J.B., Koch J.A., Deslouches B., Urish K.L. (2020). Direct antimicrobial activity of cationic amphipathic peptide WLBU2 against *Staphylococcus aureus* biofilms is enhanced in physiologic buffered saline. J. Orthop. Res..

[129] Zhang X., Zhang G., Chai M., Yao X., Chen W., Chu P.K. (2020). Synergistic antibacterial activity of physical-chemical multi-mechanism by TiO_2_ nanorod arrays for safe biofilm eradication on implant. Bioact. Mater..

[130] Li Y., Liu X., Li B., Zheng Y., Han Y., Chen D.F., Yeung K.W.K., Cui Z., Liang Y., Li Z. (2020). Near-infrared light triggered phototherapy and immunotherapy for elimination of methicillin-resistant *Staphylococcus aureus* biofilm infection on bone implant. ACS Nano.

[131] Pijls B.G., Sanders I.M.J.G., Kuijper E.J., Nelissen R.G.H.H. (2020). Synergy between induction heating, antibiotics, and *N*-acetylcysteine eradicates *Staphylococcus aureus* from biofilm. Int. J. Hyperth..

[132] Briggs T., Blunn G., Hislop S., Ramalhete R., Bagley C., McKenna D., Coathup M. (2018). Antimicrobial photodynamic therapy-a promising treatment for prosthetic joint infections. Lasers Med. Sci..

[133] Bapat P., Singh G., Nobile C.J. (2021). Visible lights combined with photosensitizing compounds are effective against *Candida albicans* biofilms. Microorganisms.

